# Episcleritis Related to Drug-Induced Lupus Erythematosus following Infliximab Therapy: A Case Report

**DOI:** 10.1155/2011/696285

**Published:** 2011-04-19

**Authors:** Irini P. Chatziralli, Evgenia Kanonidou, Alexandros Chatzirallis, Prodromos Dimitriadis, Petros Keryttopoulos

**Affiliations:** ^1^Department of Ophthalmology, General Hospital of Veroia, Veroia 59100, Greece; ^2^Medical School of Crete, University of Crete, Crete 71003, Greece; ^3^Department of Internal Medicine, General Hospital of Veroia, Veroia 59100, Greece

## Abstract

Drug-induced lupus erythematosus is defined as a lupus-like syndrome temporally related to continuous drug exposure which resolves after discontinuation of the offending drug. Herein, we describe a patient with distinct clinical manifestations of anti-TNF-associated DILE related to infliximab therapy. The patient exhibited clinical and laboratory findings of lupus-like illnesses as well as ocular disorders, such as episcleritis. The main message is that the symptoms of DILE should not be overlooked, although sometimes other systematic conditions may underlie them. As a result, it is very important for the clinicians to evaluate the symptoms of DILE and manage appropriately these cases.

## 1. Introduction

Drug-induced lupus erythematosus (DILE) is defined as a lupus-like syndrome (LLS) temporally related to continuous drug exposure which resolves after discontinuation of the offending drug. Its incidence has been estimated as up to 10% of systemic lupus erythematosus (SLE) cases [1, 2]. 

Infliximab is a human monoclonal antitumor necrosis factor (anti-TNF) agent with a good safety profile. Since the widespread usage of infliximab for the treatment of rheumatoid arthritis (RA), psoriatic arthritis (PsA), spondyloarthropathies, and inflammatory bowel disease, there have been reported several case reports of DILE and LLS associated with the use of this drug. Induction of autoantibodies in patients treated with infliximab is also well established [3–6], but ocular disorders and especially episcleritis are relatively rare.

 Herein, we describe a patient with distinct clinical manifestations of anti-TNF-associated DILE related to infliximab therapy. The patient exhibited clinical and laboratory findings of lupus-like illnesses as well as ocular disorders. These symptoms developed while receiving the anti-TNF therapy and resolved on discontinuation of the offending drug [6].

## 2. Case Presentation

A 42-year-old woman with Crohn's disease was started on infliximab in 2008 for colitis poorly responsive to conventional therapy. She had prompt and dramatic improvement in her symptoms and before the initiation of infliximab, her antinuclear antibodies (ANA) and anti-double-stranded DNA (dsDNA) were negative. One year later, she presented with polyarthritis, a rash on her face and chest, profound fatigue, and photosensitivity. Laboratory evaluation revealed an ANA titre of 1 : 160, a positive test for anti-dsDNA and for antihistone antibodies, while antiextractable nuclear antigen antibodies (ENA) were negative. The patient also presented mild thrombocytopenia and elevated CRP, whereas all other laboratory findings were normal. The diagnosis of DILE was confirmed and she stopped taking infliximab. She also received 30 mg/day prednisone for four weeks. Her symptoms gradually improved six weeks after discontinuation of infliximab and treatment with prednisone. 

Five months later, she resumed infliximab because of worsening colitis and joint pain. She developed again skin lesions, polyarthritis, and pain on eye movements, associated with acute onset redness in one eye two months after restarting infliximab. Serologic testing was positive for ANA, antihistone, and dsDNA antibodies, but remained negative for ENA. In addition to this, the patient underwent a thorough ophthalmologic examination, which revealed episcleritis. Therefore, the patient was diagnosed as a case of DILE with ocular manifestations. After stopping infliximab, the lesions faded out over the following four weeks, serologic testing normalized, and her symptoms resolved, so did the episcleritis. Written informed consent was obtained from the patient.

## 3. Discussion

As anti-TNF therapy, especially infliximab or adalimumab, becomes more widely used for a variety of diseases, the diagnosis and management for DILE will become an increasingly important challenge [3–7]. Recognition of DILE in patients receiving anti-TNF therapy can be especially difficult due to the symptoms of their underlying diseases. There are currently no standard diagnostic criteria for DILE and the pathomechanisms are still unclear. The range of symptoms which are typically present and could be employed as diagnostic criteria is confined to four (arthritis, serositis, antinuclear, and antihistone antibodies); in addition the symptoms must have begun after initiation of the treatment with a drug and must resolve on discontinuation of that drug treatment [2]. Furthermore, other symptoms may exist, such as rash, polyarthralgias, myalgias, fever, pericarditis, or photosensitivity. Central nervous system and renal involvement are usually rare [5–7]. Our patient not only had the classic symptoms of DILE but also suffered from episcleritis, which is a rare immune-mediated clinical manifestation.

Scleritis and episcleritis are well-established ocular symptoms of systematic lupus erythematosus [8–11]. Turgeon and Slamovits had described a case of scleritis in a patient using procainamide as a part of a drug-induced lupus syndrome [12]. Interestingly enough, infliximab has been used for the treatment of uveitis or scleritis [13–15]. In our case, episcleritis was not considered to be an isolated adverse effect of infliximab, but an ocular manifestation of DILE due to infliximab.

In addition, ocular manifestations of inflammatory bowel disease are not frequent, occurring in less than 10% of cases [16]. The most common ocular findings are found to be conjunctivitis, blepharitis, uveitis, episcleritis, cataract, choroiditis, retinal vasculitis, and optic neuritis [17, 18]. Our patient suffered from Crohn's disease, but she never complained of ophthalmological symptoms. Noticeably, she developed episcleritis when she was under infliximab treatment, having in parallel other DILE manifestations, and not while an active symptom period of Crohn's disease. Nevertheless, this conceptual overlap between extraintestinal manifestations of Crohn's disease and DILE should be taken into account at the interpretation of this case. 

 Laboratory abnormalities in anti-TNF DILE reported cases differ from classic DILE. Positive anti-dsDNA occurs frequently in anti-TNF-induced DILE compared with classic DILE. Antihistone antibodies are described in classic DILE more often than in anti-TNFa DILE (95% versus 57%, respectively) [1, 5, 6]. In our case, the patient had positive anti-dsDNA, ANA, and antihistone antibodies; however, ENA were negative.

Discontinuation of the treatment remains the main therapeutic intervention and most of patients experience a clinical improvement 6–12 weeks after the suppression of the implicated drug [5, 6], although there are cases that needed three weeks [7] to be improved. Our patient presented a clinical improvement after four weeks of treatment cessation.

The most astonishing feature of this case report is that anti-TNF DILE can be associated with ocular disorders, such as episcleritis. Therefore, the symptoms of DILE should not be overlooked, although sometimes other systematic conditions may underlie them. As a result, it is very important for the clinicians to evaluate the symptoms of DILE and manage appropriately these cases.

## References

[1] Marzano AV, Vezzoli P, Crosti C (2009). Drug-induced lupus: an update on its dermatologic aspects. *Lupus*.

[2] Vedove CD, Del Giglio M, Schena D, Girolomoni G (2009). Drug-induced lupus erythematosus. *Archives of Dermatological Research*.

[3] Ramos-Casals M, Brito-Zerón P, Muñoz S (2007). Autoimmune diseases induced by TNF-targeted therapies: analysis of 233 cases. *Medicine*.

[4] Mounach A, Ghazi M, Nouijai A (2008). Drug-induced lupus-like syndrome in ankylosing spondylitis treated with infliximab. *Clinical and Experimental Rheumatology*.

[5] Debandt M, Vittecoq O, Descamps V, Le Loët X, Meyer O (2003). Anti-TNF-*α*-induced systemic lupus syndrome. *Clinical Rheumatology*.

[6] Costa MF, Said NR, Zimmermann B (2008). Drug-induced lupus due to anti-tumor necrosis factor *α* agents. *Seminars in Arthritis and Rheumatism*.

[7] Martín JM, Ricart JM, Alcácer J, Rausell N, Arana G (2008). Adalimumab-induced lupus erythematosus. *Lupus*.

[8] Read RW, Weiss AH, Sherry DD (1999). Episcleritis in childhood. *Ophthalmology*.

[9] De la Maza MS, Foster CS, Jabbur NS (1995). Scleritis associated with systemic vasculitic diseases. *Ophthalmology*.

[10] Hakin KN, Watson PG (1991). Systemic associations of scleritis. *International Ophthalmology Clinics*.

[11] Frith P, Burge SM, Millard PR, Wojnarowska F (1990). External ocular finidngs in lupus erythematosus: a clinical and immunopathological study. *British Journal of Ophthalmology*.

[12] Turgeon PW, Slamovits TL (1989). Scleritis as the presenting manifestation of procainamide-induced lupus. *Ophthalmology*.

[13] Farvardin M, Afarid M, Mehryar M, Hosseini H (2010). Intravitreal infliximab for the treatment of sight-threatening chronic noninfectious uveitis. *Retina*.

[14] Jabbarvand M, Fard MA (2010). Infliximab in a patient with refractory necrotizing scleritis associated with relapsing polychondritis. *Ocular Immunology and Inflammation*.

[15] Doctor P, Sultan A, Syed S, Christen W, Bhat P, Foster CS (2010). Infliximab for the treatment of refractory scleritis. *British Journal of Ophthalmology*.

[16] Mintz R, Feller ER, Bahr RL, Shah SA (2004). Ocular manifestations of inflammatory bowel disease. *Inflammatory Bowel Diseases*.

[17] Yilmaz S, Aydemir E, Maden A, Unsal B (2007). The prevalence of ocular involvement in patients with inflammatory bowel disease. *International Journal of Colorectal Disease*.

[18] Felekis T, Katsanos K, Kitsanou M (2009). Spectrum and frequency of ophthalmologic manifestations in patients with inflammatory bowel disease: a prospective single-center study. *Inflammatory Bowel Diseases*.

